# Harnessing a Dielectric/Plasma Photonic Crystal as an Optical Microwave Filter: Role of Defect Layers and External Magnetic Fields

**DOI:** 10.3390/ma17030559

**Published:** 2024-01-24

**Authors:** Hassen Dakhlaoui, Walid Belhadj, Haykel Elabidi, Najla S. Al-Shameri, Fatih Ungan, Bryan M. Wong

**Affiliations:** 1 Nanomaterials Technology Unit, Basic and Applied Scientific Research Center (BASRC), Physics Department, College of Science of Dammam, Imam Abdulrahman Bin Faisal University, P.O. Box 1982, Dammam 31441, Saudi Arabia; 2 Physics Department, College of Sciences, Umm AL-Qura University, P.O. Box 715, Makkah 24382, Saudi Arabia; 3 Department of Physics, Faculty of Science, Sivas Cumhuriyet University, Sivas 58140, Turkey; 4 Materials Science & Engineering Program, Department of Chemistry, and Department of Physics & Astronomy, University of California-Riverside, Riverside, CA 92521, USA

**Keywords:** one-dimensional photonic crystal, transfer matrix method, transmittance spectrum, angle of incidence, plasma

## Abstract

We investigate the transmittance spectrum of a multichannel filter composed of dielectric (A) and plasma (P) materials in the microwave region within the transfer matrix formalism. Two configurations of the proposed filter are studied under the influence of an applied magnetic field: (1) a periodic structure containing ${\left(A/P\right)}^{N}$ unit cells surrounded by air and (2) the introduction of a second dielectric material (D) acting as a defect layer to produce an $(AP{)}^{N/2}/D/(AP{)}^{N/2}$ structure. Our findings reveal that in the periodic case, the number of resonant states of the transmittance increases with number N; however, the observed blue and red shifts depend on the intensity and orientation of the applied magnetic field. We present contour plots of the transmission coefficients that show the effect of the incident angle on the shifts of the photonic band gaps. Furthermore, we find that the introduction of a defect layer generates additional resonant states and merges the central resonant peak into a miniband of resonances. Moreover, we show that the number of resonant peaks and their locations can be modulated by increasing the unit cell number, N, as well as increasing the width of the inserted defect layer. Our proposed structures enable the design of novel photonic filters using magnetized plasma materials operating in the microwave region.

## 1. Introduction

The study of transmittance in photonic crystal structures containing magnetized plasma materials continues to generate significant interest due to their use in electromagnetic wave propagation and optical communication for tunable photonic filters [1,2,3]. Photonic crystals (PCs) consisting of multiple layers with a periodic refractive index can be found in one, two, and three dimensions [4,5,6]. Fabricating 1D photonic crystal structures involves employing various deposition techniques, such as chemical vapor deposition (CVD), sol–gel spin coating [7], and plasma-enhanced chemical vapor deposition (PECVD), along with radio frequency (RF) magnetron sputtering, spray pyrolysis deposition, and thermal evaporation [8]. These techniques are utilized to deposit alternating layers of dielectric materials onto a substrate, ultimately forming the periodic structure of the photonic crystal. Photolithography is then used to define the precise geometry and pattern of the layers [9]. Etching processes, such as reactive ion etching (RIE) or wet etching, are subsequently applied to selectively remove material, shaping the layers according to the designed structure. The final device may undergo additional processes like atomic layer deposition (ALD) for precise layer thickness control. Techniques such as nanoimprint lithography can be employed for nanoscale patterning.

Similar to the electronic bandgaps in semiconductor materials, PCs have photonic band gaps (PBGs) that are determined by frequencies in which the transmission of light is completely forbidden due to Bragg scattering and localized resonances [10,11,12]. Other frequencies outside the PBGs constitute allowed bands and can be transmitted across the PC structure. The PBGs in conventional PCs have been intensively studied in the context of various technological applications such as optical transistors, all-optical switches, waveguides, and optical filters [13,14,15,16,17]. PC materials are generally based on dielectrics, metals, superconductors, and semiconductors. However, plasma materials have recently been incorporated with dielectrics to construct plasma photonic crystal (PPC) structures with tunable PBGs localized in the microwave region. One can tune and control the PBGs by adjusting the plasma material’s physical properties, such as its electronic density. Recent studies have focused on the effect of external magnetic fields on PPCs, which can shift resonant states and adjust the location of the desired PBGs. The application of a magnetic field can produce a magnetized plasma photonic crystal (MPPC) with unique electromagnetic properties that are absent in conventional PCs [18,19,20,21]. For instance, Zhang and co-workers investigated the optical properties of an MPPC under the influence of magnetic fields [22]. Qi and co-workers investigated the modulation of the dispersion relation and transmission coefficient of an electromagnetic wave by varying an external magnetic field [20]. The tunability of the defect mode in a TE-polarized electromagnetic wave in a one-dimensional PC doped with a magnetized plasma was proposed by Kong et al. [23]. Furthermore, the variation observed in the PBG properties of plasma in the presence of a periodic magnetic field was studied by Bin and co-workers [24].

The majority of the previous studies addressed PBGs in MPPCs by considering the plasma layer as a defect inside a periodic conventional 1D photonic crystal; however, none of them addressed the effect of an external magnetic field on the blue and red shifts of PBGs in periodic (dielectric/plasma) structures or the effect of introducing a defect layer on the electronic transmission. The purpose of the present work is to augment the work of Li and co-workers [25] by investigating the effect of an external magnetic field and the insertion of a defect layer on the transmission coefficient in the microwave region. We demonstrate how these parameters can modulate the position of different resonant states and PBGs to aid in designing and fabricating photonic filters based on magnetized plasma materials. We provide a schematic description of the structure, a theoretical formulation for the transfer matrix method, and the dielectric constants in Section 2. Our results and their importance to the field are given in Section 3 and Section 4.

## 2. Theoretical Modeling

**Figure 1:**
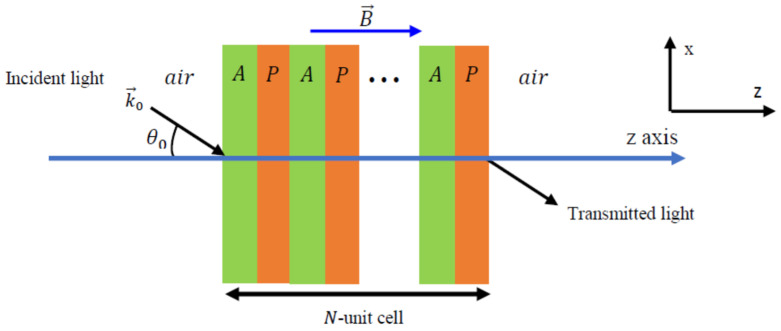
Schematic representation of the ${\left(AP\right)}^{N}$ photonic crystal structure in the presence of an applied magnetic field in the direction of the positive z-axis with an RHP configuration. *A* and *P* represent SiO_2_ and plasma layers, respectively.

We first describe a periodic structure composed of dielectric $\left(A\right)$ and plasma $\left(P\right)$ materials. The structure is surrounded by air and under the influence of an external magnetic field as shown in Figure 1. The permittivity and permeability of the plasma and dielectric layers are $\left(\varepsilon_{A},\mu_{A}\right)$ and $\left(\varepsilon_{P},\mu_{P}\right)$, respectively. The widths of the plasma and dielectric layers are $d_{A}$ and $d_{P}$, respectively. We consider an incident electromagnetic wave at an angle $\theta_{0}$ with respect to the z-axis, as shown in Figure 1. For the TM mode, the magnetic field, $\vec{B}$, is normal to the xz-plane, whereas the electric field is oriented normal to xz-plane for the TE mode. Between two consecutive layers, the magnetic and electric fields are connected by a transfer matrix that can be written as follows [25,26,27]:

(1) $$T_{i}=\left(\begin{array}{cc} \cos\left(\beta_{i}\right) & -\frac{i}{q_{i}}\sin\left(\beta_{i}\right)\\ -iq_{i}\sin\left(\beta_{i}\right) & \cos\left(\beta_{i}\right) \end{array}\right),$$

where $\beta_{i}=\frac{\omega }{c}\sqrt{\mu_{i}\varepsilon_{i}}d_{i}\;\cos(\theta_{i})$, ω is the angular frequency, ${\;d}_{i}$ is the width of the *i*th layer, $\theta_{i}$ is the angle of incidence, and c is the speed of light. The factor, $q_{i}$, is given by the following expressions for the TE and TM modes [25,26,27]:

(2) $$q_{i}=\left\{\begin{array}{c} \frac{\sqrt{\varepsilon_{i}}}{\sqrt{\mu_{i}}}\cos\left(\theta_{i}\right)\;\mathrm{for}\;\mathrm{the}\;\mathrm{TE}\;\mathrm{mode}\\ \frac{\sqrt{\mu_{i}}}{\sqrt{\varepsilon_{i}}}\cos\left(\theta_{i}\right)\;\mathrm{for}\;\mathrm{the}\;\mathrm{TM}\;\mathrm{mode} \end{array}\right.$$

The well-known transfer matrix method (TMM) was used to compute the transmittance of the structure shown in Figure 1. Our calculations were carried out with custom-developed codes in the MATLAB software environment (v2019 R) and were validated against previous studies in the scientific literature. The total matrix connecting the magnetic and electric fields of the first and last layers can be formulated as follows:

(3) E1H1=TATP……TATPEn+1Hn+1=TN=t11t12t21t22En+1Hn+1,

where $T_{A}$ and $T_{P}$ denote the transfer matrices of the dielectric and plasma layers, respectively. The elements of the total transfer matrix ${\left(T\right)}^{N}$ are $t_{11},\;t_{12},\;t_{21},$ and $t_{22}$; therefore, the coefficient of the entire system is given by [25,26,27,28,29,30]

(4) $$t_{C}=\frac{2q_{0}}{t_{11}q_{0}+t_{12}q_{0}q_{n+1}+t_{21}+t_{22}q_{n+1}},$$

where $q_{n+1}$ and $q_{0}$ represent the last and first medium. The transmission coefficient is given by

(5) $$T_{C}=\frac{q_{n+1}}{q_{0}}{\left|t_{C}\right|}^{2}.$$

The dielectric coefficients of the plasma layers under the applied magnetic field depend on the frequency and are given by the following expressions [18,19,20,22,23,24,30,31,32,33,34]:

(6) $$\varepsilon_{p}^{R}\left(\omega ,B\right)=\left\{1-\frac{\varepsilon_{pe}^{2}(\omega -\omega_{le})}{\omega \left[{\left(\omega -\omega_{le}\right)}^{2}+\gamma^{2}\right]}\right\}-j\left\{\frac{{\gamma \varepsilon }_{pe}^{2}}{\omega \left[{\left(\omega -\omega_{le}\right)}^{2}+\gamma^{2}\right]}\right\},$$

(7) $$\varepsilon_{p}^{L}\left(\omega ,B\right)=\left\{1-\frac{\varepsilon_{pe}^{2}(\omega +\omega_{le})}{\omega \left[{\left(\omega +\omega_{le}\right)}^{2}+\gamma^{2}\right]}\right\}-j\left\{\frac{{\gamma \varepsilon }_{pe}^{2}}{\omega \left[{\left(\omega +\omega_{le}\right)}^{2}+\gamma^{2}\right]}\right\}.$$

where $\omega ,\;B,\;\mathrm{and}\omega_{pe}$ are the angular frequency of light, applied magnetic field, and plasma frequency, respectively. The plasma frequency depends on the electron mass $\left(m\right)$, charge $\left(e\right)$, and density according to the following expression:

(8) $$\omega_{pe}=\sqrt{\frac{n_{e}^{2}\;e}{m\varepsilon_{0}}},$$

where $\omega_{le}$ is a function of the magnetic field $\left(B\right)$, charge $\left(e\right)$, electron mass $\left(m\right)$ and is calculated using $\omega_{le}=\frac{eB}{m}$. The superscripts L and R denote left-hand polarized (LHP) and right-hand polarized (RHP) configurations, respectively. The RHP configuration is obtained when the magnetic field is aligned with the positive *z* direction, whereas the LHP configuration occurs when the magnetic field is oriented towards negative *z*. The numerical values of the physical parameters used in this investigation are [22,33] $\omega_{pe}=2\pi \times 10^{9}\;\mathrm{Hz}$; $\gamma =4\pi \times 10^{4}\;\mathrm{Hz}$; $n_{e}=8\times 10^{17}\;m^{-3}$, and the widths of the dielectric and plasma layers are fixed at $d_{A}=d_{P}=7\;\mathrm{mm}$. The SiO_2_ dielectric layer, denoted by *A*, has a dielectric constant of $\varepsilon_{A}=4$, and the BaTiO_3_ defect layer, represented by *D*, has a dielectric constant of $\varepsilon_{D}=5.8$ [35,36]. The plasma layers are denoted by *P*. These structures can be realized using a sol–gel spin-coating technique [7]. The magnetic field $\left(B\right)$, number of unit cells $\left(N\right)$, angle of incidence $\left(\theta_{0}\right)$, and width of the defect layer $\left(d_{D}\right)$ are taken as variables in our simulations to study their effect on the resonant states and PBGs produced by our photonic filter.

## 3. Results and Discussion

### 3.1. Periodic Structure under Magnetic Field

In this section, we consider a periodic photonic crystal structure, $(AP{)}^{N}$, under the influence of an external magnetic field. The structure is surrounded by air and illuminated by light at an angle of incidence, $\theta_{0}$. We study the effect of the parameters $\theta_{0},\;N,\;\mathrm{and}\;B$ on the transmission coefficient and the PBGs.

**Figure 2:**
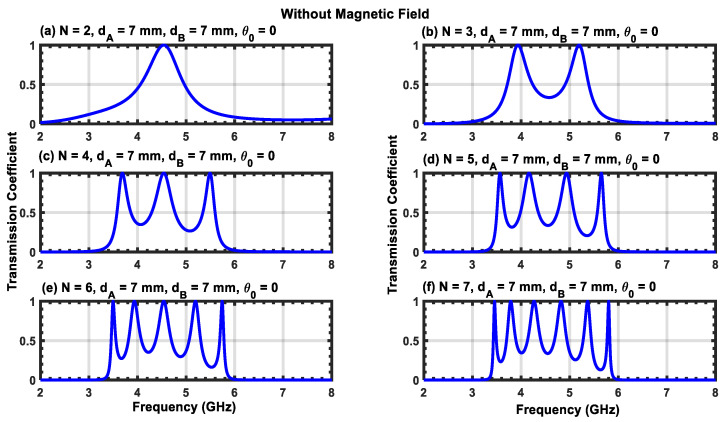
(**a**–**f**) Transmission coefficients for normal incidence in the absence of an applied magnetic field. The parameters for the simulation are given at the top of each subfigure.

Figure 2a–f show the transmission coefficient as a function of frequency for N=2, 3, 4, 5, 6,and 7 with the magnetic field turned off (B=0). For the case of normal incidence (θ=0), the transmission coefficients display sharper peaks corresponding to the resonant frequencies. The number of resonant peaks is equal to N−1, and their existence is a consequence of the interaction between the incident and evanescent waves in the plasma layers. For N=2, one peak is centered at a frequency of f=4.5 GHz with a perfect transmission $\left(T_{C}=1\right)$. This peak remains unaffected for all even values of N, whereas it becomes a valley in the case of odd values. As N increases, the interval between the different peaks is significantly reduced such that the peaks assemble together to form a band pass filter [18,30]. In addition, we observed that the resonant peaks become sharper at higher values of N, and the interval between consecutive peaks is significantly reduced. This change can be used to obtain a miniband of resonances to design an optical filter with multiple channels.

**Figure 3:**
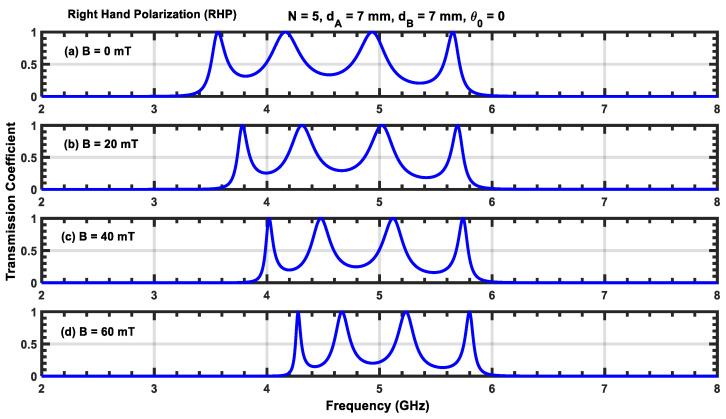
(**a**–**d**) Transmission coefficient for N=5 at normal incidence for different values of B with RHP polarization. (**a**) B=0 mT; (**b**) B=20 mT; (**c**) B=40 mT; (**d**) B=60 mT.

**Figure 4:**
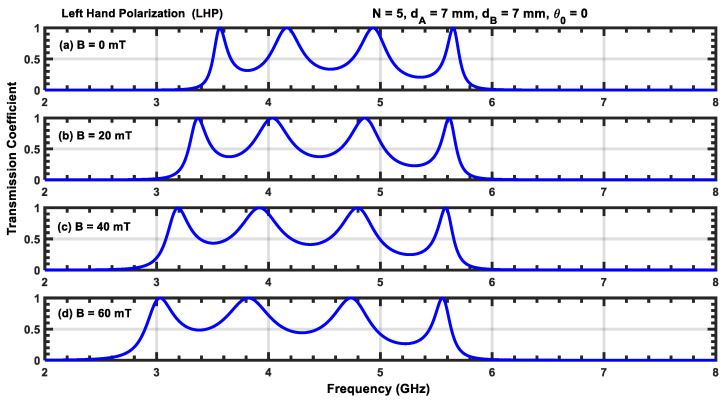
(**a**–**d**) Transmission coefficient for N=5 at normal incidence for different values of B with LHP polarization. (**a**) B=0 mT; (**b**) B=20 mT; (**c**) B=40 mT; (**d**) B=60 mT.

The transmission coefficient for a structure containing five periods $\left(N=5\right)$ for four different values of the applied magnetic field with RHP and LHP polarizations is given in Figure 3 and Figure 4. For RHP polarizations, the resonant peaks move toward higher frequencies as the magnetic field intensity is increased (i.e., the resonant states blue shift as B increases). However, for RHP polarizations, the resonant states move to lower frequencies, resulting in a red shift. 

Furthermore, the displacement of the resonant peak at the lower resonant frequency moves more rapidly than the peak at the higher resonant frequency for both RHP and LHP polarizations. This is due to the strong dependence on the plasma permittivity, which can be seen in Equations (6) and (7). In addition, an increase in B makes the resonant peaks sharper in the RHP polarization, while these peaks are enlarged for LHP polarization. Increasing B also reduces the frequency interval between consecutive resonant peaks for RHP polarizations but increases it for LHP polarizations (i.e., increasing the magnetic field expands different channels during RHP polarization but squeezes them during LHP polarization). This phenomenon can be leveraged to control optical filters based on magnetized plasma photonic crystals by adjusting the orientation of the applied magnetic field.

**Figure 5:**
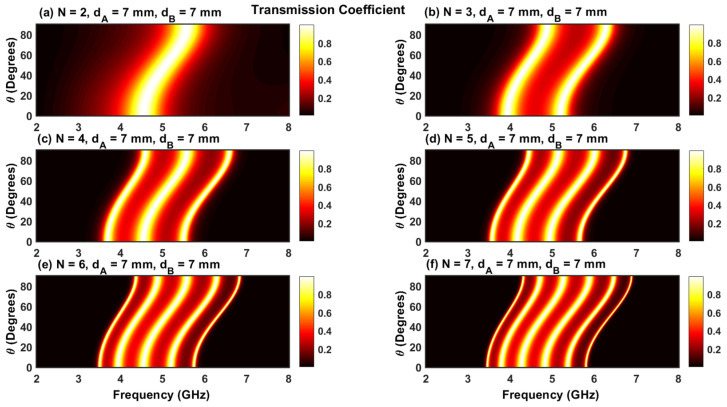
(**a**–**f**) Contour plot of the transmission coefficient as a function of the frequency and angle of incidence without magnetic field. The parameters of each simulation are given at the top of each subfigure.

To understand the effect of the angle of incidence, Figure 5a–f show the transmission coefficient as a function of θ and frequency. It is apparent that all transmissions contain permitted bands surrounded by PBGs. The number of permitted bands in each figure is equal to N−1, which confirms the results mentioned previously in Figure 2. Upon increasing the angle of incidence, the permitted bands move to larger frequencies. In addition, Figure 5a–f clearly show that the widths of the permitted bands are reduced after increasing N. In addition, we observed that the shift of the permitted bands is less sensitive to the change in incident angles near θ=0 and π/2. As such, the most effective way to tune the permitted bands towards higher frequencies is to emit light with incident angles between 20 and 80 degrees. 

**Figure 6:**
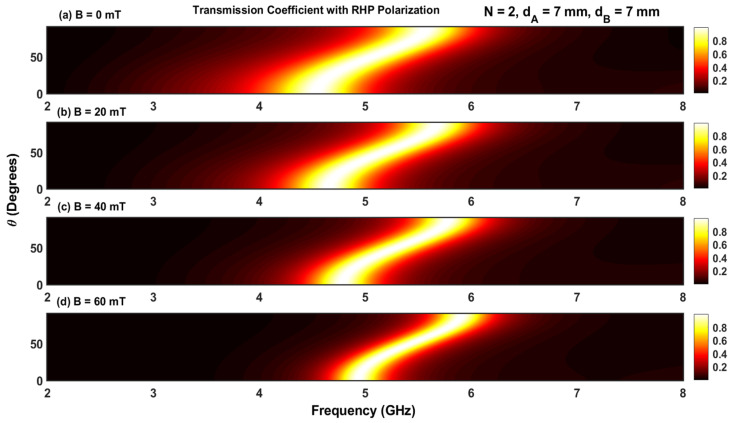
(**a**–**d**) Contour plot of the transmission coefficient with RHP polarization as a function of frequency and angle of incidence.

**Figure 7:**
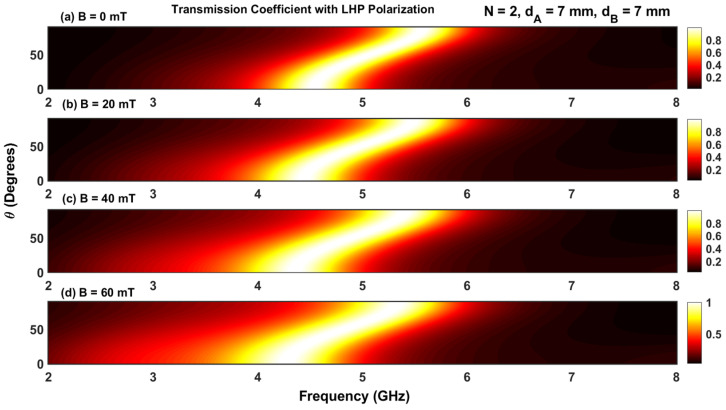
(**a**–**d**) Contour plot of the transmission coefficient with LHP polarization as a function of frequency and angle of incidence.

The effect of the magnetic field with LHP and RHP polarizations on the transmission coefficients for different angles of incidence is shown in Figure 6a–d and Figure 7a–d. The simulations were carried out for N=2 and four values of magnetic fields (B=0, B=20, B=40, and B=60 mT). The contour plots in Figure 6 and Figure 7 contain only one permitted band, which is in accordance with the result shown in Figure 2a. Increasing the magnetic field introduces a shift of the permitted band toward higher and lower frequencies for RHP and LHP polarizations, respectively. Furthermore, at a given value of the applied magnetic field, the variation in the permitted band is negligible for lower and higher angles of incidence (θ=0 and π/2).

### 3.2. Effects of a Defect Layer on the Resonant States

**Figure 8:**
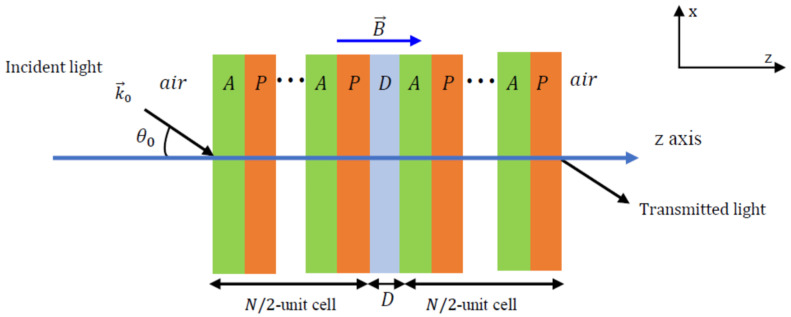
Schematic representation of the $(AP{)}^{N/2}/D/(AP{)}^{N/2\;}$ photonic crystal structure in the presence of an applied magnetic field in the direction of the positive *z*-axis. A, P, and D represent the SiO_2_, plasma layer, and BaTiO_3_, respectively.

In this section, we consider a defect layer, D, incorporated in the previous $(AP{)}^{N}$ periodic structure under the action of an external magnetic field. The new structure is ($AP{)}^{N/2}/D/(AP{)}^{N/2}$, as shown in Figure 8. As before, the crystal is surrounded by air and illuminated by light at an angle of incidence $\theta_{0}$. 

**Figure 9:**
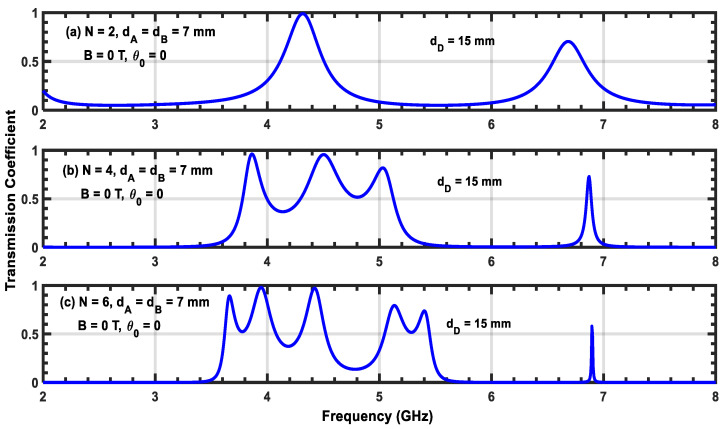
(**a**–**c**) Transmission coefficient of the $(AP{)}^{N/2}/D/(AP{)}^{N/2}$ structure with normal incidence without a magnetic field. The width of the defect layer is $d_{D}=15\;\mathrm{mm}$.

**Figure 10:**
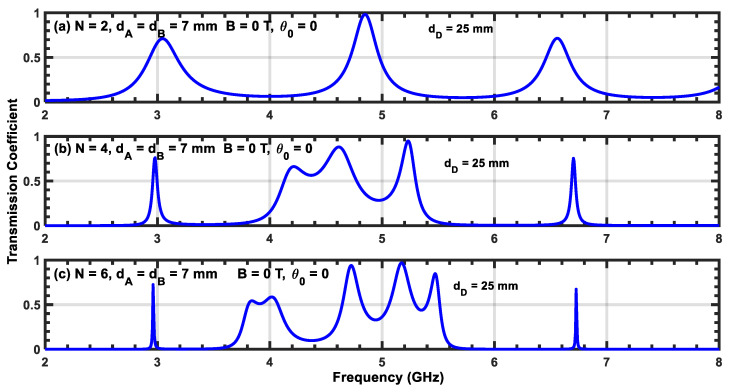
(**a**–**c**) Transmission coefficient of the $(AP{)}^{N/2}/D/(AP{)}^{N/2}$ structure with normal incidence without a magnetic field. The width of the defect layer is $d_{D}=25\;\mathrm{mm}$.

**Figure 11:**
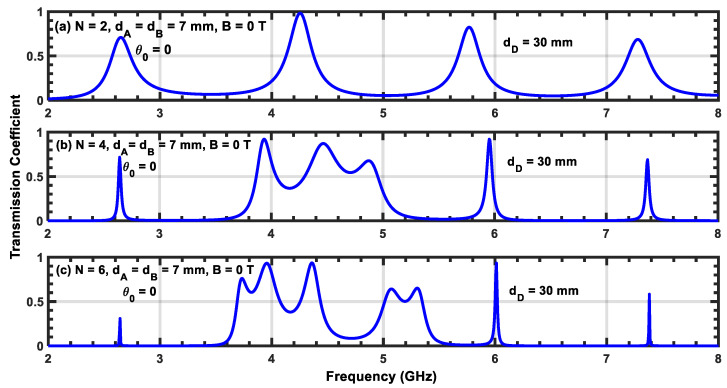
(**a**–**c**) Transmission coefficient of the ($(AP{)}^{N/2}/D/(AP{)}^{N/2}$ structure with normal incidence without a magnetic field. The width of the defect layer is $d_{D}=30\;\mathrm{mm}$.

Figure 9a–c, Figure 10a–c, and Figure 11a–c show transmission coefficients at normal incidence as a function of frequency for three different widths, $d_{D}$, of the defect layer: 15, 25, and 30 mm, respectively. In all of these cases, the magnetic field is turned off. Figure 9a shows two resonant peaks located at $f_{1}=4.3\;\mathrm{GHz}$ and $f_{2}=6.7\;\mathrm{GHz}$. Comparing this figure with Figure 2a, which contains one resonant state, we show that the addition of the defect layer creates a secondary peak with a low transmission value at frequency $f_{2}$. Upon increasing N from 2 to 6, we observe that the resonant peak corresponding to the first frequency $f_{1}$ splits into additional peaks, and we obtain a permitted miniband of frequencies containing peaks and valleys. In addition, the number of these secondary resonant peaks increases with N. Furthermore, we observe that the peak corresponding to frequency $f_{1}$ becomes sharper and moves slightly toward higher frequencies (i.e., a blue shift). Upon increasing the value of $d_{D}$, the structure exhibits additional resonant states, and the central peak located at $f_{1}$ generates more secondary peaks. Therefore, the inserted defect layer plays an important role in producing multiple channels in the transmission coefficient of the structure. However, increasing N shifts the position of each resonant peak to higher frequencies and reduces their half-widths. Since the reduction in the half-width of any resonant state affects the sensitivity of the structure, this can be leveraged to design sensor devices based on plasma photonic crystals.

**Figure 12:**
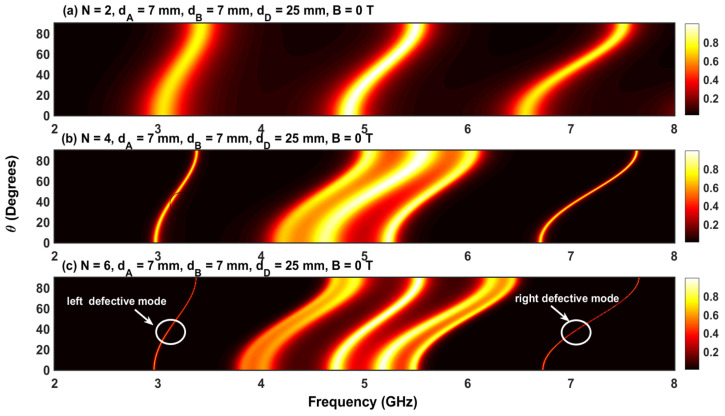
(**a**–**c**) Transmission coefficient in a ${\left(AP\right)}^{N/2}D{\left(AP\right)}^{N/2}$ structure at normal incidence without a magnetic field. The width of the defect layer is $d_{D}=25\;\mathrm{mm}$.

To understand the simultaneous effect of both incident frequency and angles of incidence, Figure 12a–c show contour plots of the transmission coefficient as a function of θ and frequency without a magnetic field. The width of the defect layer is fixed at $d_{D}=25\;\mathrm{mm}$. The transmission coefficient shows PBGs when the angle of incidence is increased. At normal incidence (θ=0), we observe the three resonant states previously observed in Figure 10a. One central resonant state and two secondary resonant states representing the right and left defect modes arise due to the insertion of the defect layer, D. Upon augmenting θ, the resonant states move toward higher frequencies (i.e., a blue shift). Furthermore, the slope of the frequencies delimiting these resonant states is not linear at lower (θ≤20°) and higher angles (θ≥80°) of incidence. In addition, upon increasing the number of periods, N, the central resonant state generates new secondary resonant states, resulting in a permitted miniband region. However, the two resonant states corresponding to the defect layer become sharper and increase in intensity as N increases, a characteristic that can be leveraged for the design of optical filters, as mentioned previously.

**Figure 13:**
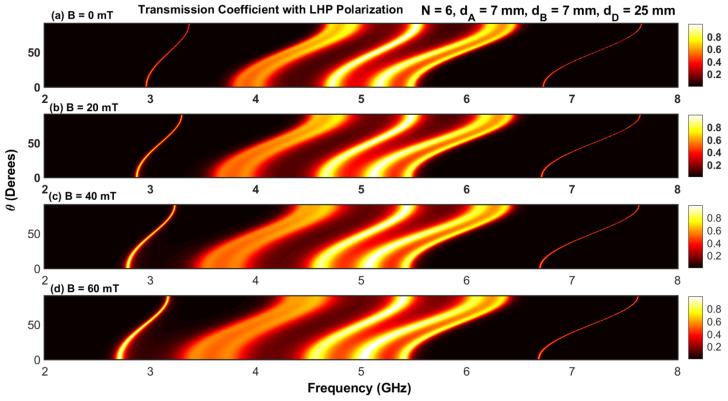
(**a**–**d**) Transmission coefficient in ${\left(AP\right)}^{N/2}D{\left(AP\right)}^{N/2}$ structure at normal incidence with a magnetic field (LHP). The width of the defect layer is $d_{D}=25\;\mathrm{mm}$.

**Figure 14:**
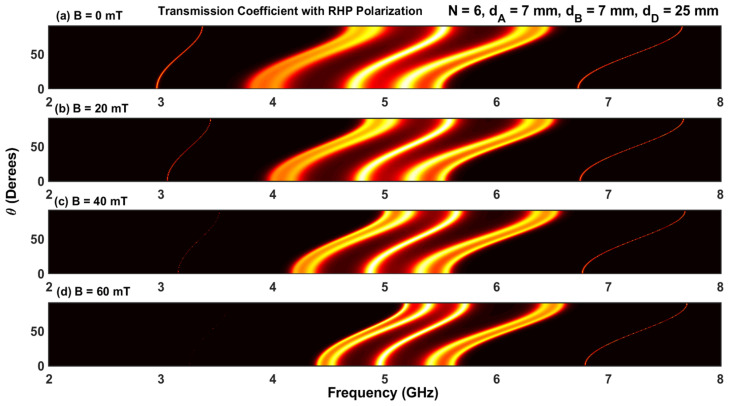
(**a**–**d**) Transmission coefficient in ${\left(AP\right)}^{N/2}D{\left(AP\right)}^{N/2}$ structure at normal incidence with a magnetic field (RHP). The width of the defect layer is 25 mm.

The effect of the magnetic field on the resonant states and photonic bandgaps is shown in Figure 13a–d and Figure 14a–d for both the LHP and RHP polarizations. We considered four values of the applied magnetic field: B=0, 20, 40, and 60 mT. The number of unit cells and width of the defect layer is N=6, and $d_{D}=25\;\mathrm{mm}$, respectively. Upon comparing the results in these figures with those in Figure 12, we found that the application of the magnetic field transforms the unique central resonant state into a central permitted miniband of frequencies. Upon increasing the intensity of the magnetic field, the frequencies delimiting this band move toward lower values for the LHP polarization. While the frequency of the left defect mode moves toward lower values, the frequency of the right defect mode remains unchanged. However, in the case of RHP polarization, the frequency of the left defect mode moves toward higher values, with the frequency corresponding to the right defect mode being practically unchanged. As shown in Figure 14a–d, the amplitude of the right defect is significantly reduced when the magnetic field intensity is increased and is completely suppressed for B≥40 mT. On the other hand, the amplitude and frequency of the right defect mode are not affected by increasing B. As such, the defect modes that appear by applying an external magnetic field can play an important role in the design of an optical filter since they produce additional resonant states completely separate from the central miniband, and their amplitudes can be modulated by the angle of incidence, frequency, and magnetic field intensity.

## 4. Conclusions

In conclusion, we investigated the transmission of light across a magnetized plasma-based 1D photonic crystal with and without a defect layer. The transfer matrix method was employed to calculate the transmission coefficient for a different number of unit cells and polarizations of the magnetic field. In the absence of a defect layer, the transmission coefficient displays *N* − 1 resonant states separated by photonic bandgaps. The application of a magnetic field displaces the resonant peaks towards lower and higher frequencies for RHP and LHP polarizations, respectively. In addition, we investigated the effect of an inserted defect layer on the transmission coefficient and found that an increase in the defect layer width for a fixed number of unit cells, N, introduces additional resonant states. However, increasing N for a given defect layer width produces additional resonant states around the central state with a permitted miniband of frequencies. Our findings for modulating the photonic properties of these novel materials can be useful in designing and fabricating new magnetized devices such as optical reflectors, microwave antennae, and filters, particularly in the microwave region of the electromagnetic spectrum.

## Data Availability

Data are contained within the article.
